# The bisphosphonate, zoledronic acid, induces apoptosis of breast cancer cells: evidence for synergy with paclitaxel

**DOI:** 10.1054/bjoc.2001.1727

**Published:** 2001-04

**Authors:** S P Jagdev, R E Coleman, C M Shipman, A Rostami-H, P I Croucher

**Affiliations:** YCR Department of Clinical Oncology, Division of Biochemical and Musculoskeletal Medicine, Weston Park Hospital Sheffield, University of Sheffield Medical School, Sheffield, UK

**Keywords:** breast cancer, bisphosphonate, apoptosis, paclitaxel

## Abstract

Bisphosphonates are well established in the management of breast-cancer-induced bone disease. Recent studies have suggested that these compounds are effective in preventing the development of bone metastases. However, it is unclear whether this reflects an indirect effect via an inhibition of bone resorption or a direct anti-tumour effect. The breast cancer cell lines, MCF-7 and MDA-MB-231 cells were treated with increasing concentrations of the bisphosphonate, zoledronic acid, for varying time periods, in the presence or absence of paclitaxel. The effects of zoledronic acid were determined by assessing cell number and rate of apoptosis by evaluating changes in nuclear morphology and using a fluorescence nick translation assay. Zoledronic acid caused a dose- and time-dependent decrease in cell number (*P*< 0.001) and a concomitant increase in tumour cell apoptosis (*P*< 0.005). Short-term exposure to zoledronic acid was sufficient to cause a significant reduction in cell number and increase in apoptosis (*P*< 0.05). These effects could be prevented by incubation with geranyl geraniol, suggesting that zoledronic acid-induced apoptosis is mediated by inhibiting the mevalonate pathway. Treatment with zoledronic acid and clinically achievable concentrations of paclitaxel resulted in a 4–5-fold increase in tumour cell apoptosis (*P*< 0.02). Isobologram analysis revealed synergistic effects on tumour cell number and apoptosis when zoledronic acid and paclitaxel were combined. Short-term treatment with zoledronic acid, which closely resembles the clinical setting, has a clear anti-tumour effect on breast cancer cells. Importantly, the commonly used anti-neoplastic agent, paclitaxel, potentiates the anti-tumour effects of zoledronic acid. These data suggest that, in addition to inhibiting bone resorption, zoledronic acid has a direct anti-tumour activity on breast cancer cells in vitro. © 2001 Cancer Research Campaign http://www.bjcancer.com


*British Journal of Cancer* (2001) **84**(8), 1126–1134
© 2001 Cancer Research Campaign
doi: 10.1054/ bjoc.2001.1727, available online at http://www.idealibrary.com on IDEAL®

### The bisphosphonate, zoledronic acid, induces apoptosis of breast cancer cells: evidence for synergy with paclitaxel

**SP Jagdev, RE Coleman, CM Shipman, A Rostami-H and PI Croucher**

YCR Department of Clinical Oncology, Weston Park Hospital, Sheffield and the Division of Biochemical and Musculoskeletal Medicine, University of Sheffield Medical School, Sheffield, UK

**Summary** Bisphosphonates are well established in the management of breast-cancer-induced bone disease. Recent studies have suggested that these compounds are effective in preventing the development of bone metastases. However, it is unclear whether this reflects an indirect effect via an inhibition of bone resorption or a direct anti-tumour effect. The breast cancer cell lines, MCF-7 and MDA-MB-231 cells were treated with increasing concentrations of the bisphosphonate, zoledronic acid, for varying time periods, in the presence or absence of paclitaxel. The effects of zoledronic acid were determined by assessing cell number and rate of apoptosis by evaluating changes in nuclear morphology and using a fluorescence nick translation assay. Zoledronic acid caused a dose- and time-dependent decrease in cell number ($P < 0.001$) and a concomitant increase in tumour cell apoptosis ($P < 0.005$). Short-term exposure to zoledronic acid was sufficient to cause a significant reduction in cell number and increase in apoptosis ($P < 0.05$). These effects could be prevented by incubation with geranyl geraniol, suggesting that zoledronic acid-induced apoptosis is mediated by inhibiting the mevalonate pathway. Treatment with zoledronic acid and clinically achievable concentrations of paclitaxel resulted in a 4–5-fold increase in tumour cell apoptosis ($P < 0.02$). Isobologram analysis revealed synergistic effects on tumour cell number and apoptosis when zoledronic acid and paclitaxel were combined. Short-term treatment with zoledronic acid, which closely resembles the clinical setting, has a clear anti-tumour effect on breast cancer cells. Importantly, the commonly used anti-neoplastic agent, paclitaxel, potentiates the anti-tumour effects of zoledronic acid. These data suggest that, in addition to inhibiting bone resorption, zoledronic acid has a direct anti-tumour activity on breast cancer cells in vitro. © 2001 Cancer Research Campaign http://www.bjcancer.com

**Keywords**: breast cancer; bisphosphonate; apoptosis; paclitaxel

In recent years bisphosphonates have become important in the management of cancer-induced bone disease and are now well established in the management of patients with multiple myeloma and bone metastases secondary to breast cancer (Paterson et al, 1993; Berenson et al, 1996; Hortobagyi et al, 1996; Diel et al, 1998; McCloskey et al, 1998; Theriault et al, 1999). Randomized controlled trials in patients with breast cancer have demonstrated that clodronate and pamidronate reduce skeletal morbidity and improve quality of life (Paterson et al, 1993; Hortobagyi et al, 1996; Kanis et al, 1996). Furthermore, in the adjuvant situation, studies with oral clodronate suggest that the use of bisphosphonates in combination with chemotherapy or endocrine therapy may delay first recurrence in bone (Diel et al, 1998). In addition, Diel et al have shown that treatment with oral clodronate may be associated with reduced rates of visceral metastases and improved survival compared with those receiving standard antineoplastic therapy alone (Diel et al, 1998). However, the role of bisphosphonates in the adjuvant setting remains to be clarified as Saarto et al reported an increase in the frequency of bone and visceral metastases with poorer survival in clodronate treated patients when compared with control (Saarto et al, 2000).

*Received 14 June 2000*
*Received 23 January 2001*
*Accepted 30 January 2001*

*Correspondence to:* SP Jagdev

The mechanisms by which bisphosphonates elicit their anti-resorptive activity are only now becoming clear. Recent data suggest that bisphosphonates that are similar in structure to pyrophosphate, such as clodronate and etidronate, are metabolized to cytotoxic analogues of ATP (Frith et al, 1997) and these analogues are responsible for inhibiting osteoclast activity. In contrast, nitrogen-containing bisphosphonates, which include pamidronate, ibandronate, alendronate and zoledronic acid (Zometa®), induce apoptosis in osteoclasts by inhibiting enzymes of the mevalonate pathway and preventing the isoprenylation of small GTP-binding proteins such as Ras and Rho (Hughes et al, 1995; Luckman et al, 1998). However, bisphosphonates have also been shown to have effects on cells other than osteoclasts, including tumour cells. Shipman et al have demonstrated that the nitrogen-containing bisphosphonate, incadronate, could induce apoptosis of human multiple myeloma cells in vitro (Shipman et al, 1997), an effect that was mediated by inhibition of the mevalonate pathway (Shipman et al, 1998). Studies have also suggested that bisphosphonates can inhibit the adhesion of breast cancer cells to bone matrices (Van der Pluijm et al, 1996; Boissier et al, 1997; Magnetto et al, 1999) and can enhance the ability of anti-neoplastic agents to inhibit breast cancer cell invasion (Magnetto et al, 1999). Thus, the demonstration that these compounds can induce apoptosis in cells other than osteoclasts, and that the treatment of patients with bisphosphonates may improve survival, raises the intriguing possibility that bisphosphonates may also have direct anti-tumour effects in breast cancer cells. The aim of the present studies was to determine whether the third generation bisphosphonate zoledronic acid has direct anti-tumour effects on breast cancer cells in vitro and, more importantly, whether zoledronic acid has additive or synergistic effects with anti-neoplastic agents, such as paclitaxel, at clinically relevant concentrations.

### MATERIALS AND METHODS

### Chemicals

Zoledronic acid 1-hydroxy-2-[(1H-imidazol-1-yl) ethylidene] bisphosphonic acid monohydrate was supplied as the hydrated disodium salt by Novartis Pharma AG (Basel, Switzerland). A stock solution was prepared in phosphate-buffered saline (PBS) (pH 7.4) and filter-sterilized using a 0.22-μM filter (Gelman Sciences, Northampton, UK). Farnesol (FOH) and geranylgeraniol (GGOH) were purchased from Sigma Chemical Co (Poole, UK) and American Radiolabeled Chemicals Inc (St Louis, MO), respectively. Stock solutions were prepared in 100% ethanol. All other chemicals, unless otherwise stated, were obtained from Sigma Chemical Co.

### Cell culture

The human breast carcinoma cell line MCF-7 was obtained from the European Collection of Animal Cell Cultures (Salisbury, UK). Professor R Nicholson (University of Cardiff, Wales, UK) kindly provided the MDA-MB-231 human breast carcinoma cell line. Cells were cultured in RPMI 1640 (Life Technologies, Inc, Paisley, UK) supplemented with 10% FCS, 1 mM glutamine, 1 mM sodium pyruvate and 1x MEM nonessential amino acids (Life Technologies Inc). Cells were harvested using trypsin/ETDA 0.5%/0.2% (Life Technologies).

### Measurement of cell number

MCF-7 and MDA-MB-231 breast cancer cells were plated at a density of $5 \times 10^4$ cells $ml^{-1}$ in 24-well plates in replicates of 4 wells per treatment and allowed to adhere and proliferate for 72 hours. Cells were then incubated with fresh media containing increasing concentrations of zoledronic acid (0.1–100 μM) for 72 hours. In some studies, MCF-7 cells were seeded as described above and incubated with 100 μM zoledronic acid for 24, 48, 72 and 96 hours. MCF-7 cells were also incubated with 100 μM EDTA for 72 hours to determine the effects of calcium chelation on cell number. In experiments to determine whether there was a synergistic effect when paclitaxel and zoledronic acid were combined, MCF-7 cells were incubated in the presence or absence of 10 μM zoledronic acid and/or 2 nM paclitaxel acid for a total of 72 hours. Cells were also incubated with increasing concentrations of paclitaxel alone and in combination with increasing concentrations of zoledronic acid for a total of 72 hours to enable the construction of isobolograms. In order to assess the effects of acute exposure to zoledronic acid, MCF-7 cells were prepared as described above and incubated with or without zoledronic acid for 2, 6 or 24 hours. The media was then replaced with fresh media without zoledronic acid and the cells incubated for a total of 72 hours. In each experiment, following incubation, cells were harvested, suspended in PBS and cell number determined using a haemocytometer (Improved Neubauer).

### Identification of apoptotic cells by examination of nuclear morphology

MCF-7 and MDA-MB-231 breast cancer cells were cultured as described above for assessment of nuclear morphology with 4 replicates per treatment. Cells were incubated with zoledronic acid as above, harvested, and fixed with 4% formaldehyde (v/v) in PBS and cytospun onto glass slides. Nuclei were stained with 4′-6-diamidino-2-phenyl-indole (DAPI) at 2 μg $ml^{-1}$ in PBS for 15 minutes and examined, using a DMRB fluorescence microscope (Leica), for the characteristic changes of apoptosis (Wyllie et al, 1980).

### Identification of apoptotic cells by the in situ nick translation assay

MCF-7 and MDA-MB-231 breast cancer cells were seeded at $1 \times 10^5$ cells $ml^{-1}$ in 6-well plates, allowed to adhere and proliferate for 72 hours and then treated as described in the previous section. In some experiments, MCF-7 cells were treated with 100 μM zoledronic acid in the presence or absence of mevalonate, 50 μM farnesol or 50 μM geranylgeraniol, for 72 hours, in order to determine the effects of intermediates of the mevalonate pathway on zoledronic acid-induced apoptosis. Cells were harvested, washed in sterile PBS, fixed in 4% formaldehyde for 15 minutes on ice and permeabilized by incubation with 100% ethanol overnight. To label DNA strand breaks in apoptotic cells, the samples were incubated at room temperature with a buffer containing 50 mM tris, 2.5 mM magnesium chloride, 10 μg $ml^{-1}$ bovine serum albumen, 10 mM β-mercaptoethanol, 0.2 nM of unlabelled dATP, dGTP and dCTP and 0.2 nM biotin-16-dUTP in the presence of 1 unit DNA polymerase for 4 hours with intermittent gentle agitation (Gorczyca et al, 1993). The cells were then washed in sterile PBS and incubated with a second buffer containing 4× saline-sodium citrate buffer (SSC), 0.1% Triton-X 100, 5% (w/v) fat-free powdered milk and 1% (v/v) phycoerythrin-conjugated streptavidin (DAKO) for 30 minutes at room temperature in the dark. After washing with PBS, cells were analysed using flow cytometry.

### Statistical analysis

Results were expressed as mean ± SEM. The relationship between the concentration of zoledronic acid, time of incubation with zoledronic acid, and effects on cell number and apoptosis were analysed using the non-parametric Kruskal–Wallis Analysis by Rank test. Experiments using the in situ nick translation assay were performed with a single sample per treatment. These data were therefore not analysed for statistical significance. All other data were analysed using non-parametric Mann–Whitney *U* test. All experiments were repeated on at least 2 separate occasions unless specified otherwise.

### Isobologram analysis

The isobologram method of analysis was used to assess whether synergistic effects were seen when paclitaxel and zoledronic acid were combined. This method allows the identification of interactions between 2 drugs, regardless of mechanism of action of the individual drug (Berenbaum, 1989; Greco et al, 1995). Dose-response curves were plotted for the effects of paclitaxel and the

*© 2001 Cancer Research Campaign*

effects of zoledronic acid on MCF7 cell number. From these, the combined drug $IC_{75}$ values (75% of maximum effect, 25% remaining) were determined for each curve. The ratio of the combined drug $IC_{75}$ to the $IC_{75}$ value of each drug along was calculated and plotted as an isobologram (D/D75). This analysis was repeated for the data obtained from experiments determining the effect of paclitaxel and zoledronic acid on apoptosis. In this case, $IC_5$ values and the corresponding ratios (D/D5) were calculated from the dose–response curves in order to maximize the number of data points on the isobologram.

### RESULTS

### Zoledronic acid causes a reduction in breast cancer cell number

Treatment of MCF-7 breast cancer cells with increasing concentrations of zoledronic acid for 72 hours caused a dose-dependent decrease in cell number (Figure 1A) ($P < 0.001$). 0.1–1 μM zoledronic acid had little effect on the number of MCF-7 cells. In contrast, zoledronic acid at 10 and 100 μM caused a significant reduction in the proportions of MCF-7 cells (49.54 ± 5.43% and 23.55 ± 5.43% of control, respectively) ($P < 0.05$). Zoledronic acid had little effect on MDA-MB-231 cells at concentrations of 0.1–10 μM, whereas the 100 μM concentration resulted in a significant reduction in cell number (25.33 ± 6.0% of control) ($P < 0.05$). Treatment of breast cancer cells with 100 μM zoledronic acid for increasing periods of time resulted in a 63.5% reduction in MCF-7 cell number at 72 hours ($6.8 \pm 5.3 \times 10^4$ vs $18.6 \pm 3.2 \times 10^4$) and an 87.1% reduction at 96 hours ($3.81 \pm 1.9 \times 10^4$ vs $29.6 \pm 4.5 \times 10^4$) ($P < 0.05$ in each case) (Figure 1B). Incubation of MCF-7 cells with 100 μM EDTA resulted in a small but significant reduction in cell number when compared with control ($16.08 \times 10^4 \pm 4.2$ vs $24.52 \times 10^4 \pm 4.3$). However, treatment with zoledronic acid caused a greater reduction in cell number ($7.00 \times 10^4 \pm 1.5$ vs $24.52 \times 10^4 \pm 4.3$) and this was significantly lower than that caused by EDTA ($P < 0.05$ when zoledronic acid was compared to EDTA) (Table 1).

### Zoledronic acid induces apoptosis of breast cancer cells

Treatment of MCF-7 and MDA-MB-231 cells with zoledronic acid caused an increase in the proportion of cells with nuclear morphology characteristic of apoptosis. These characteristics included nuclear fragmentation, chromatin condensation and the formation of dense, rounded apoptotic bodies (Figure 2). Treatment of cells with increasing concentrations of zoledronic acid (0.1–100 μM) resulted in a dose-dependent increase in the proportion of cells undergoing apoptosis ($P < 0.005$). Concentrations of 0.1 and 1 μM zoledronic acid had little effect on MCF-7 cell apoptosis. In contrast, treatment with 10 μM zoledronic acid resulted in a greater than 4-fold increase in MCF-7 cell apoptosis (431.26 ± 32.21% of control) whereas the 100 μM concentration induced a 6-fold increase in the proportion of apoptotic cells (652.54 ± 71.49% of control) (Figure 2C). Treatment of MDA-MB-231 cells with concentrations of 0.1 μM, 1 μM and 10 μM bisphosphonate had little effect on apoptosis. However, MDA-MB-231 cells showed a 15-fold increase in apoptosis (1515.24 ± 139.33% of control) after treatment with 100 μM zoledronic acid ($P < 0.05$). Treatment of MCF-7 cells with 100 μM zoledronic acid for 24–96 hours caused a significant time-dependent increase in apoptosis ($P < 0.005$) (Figure 2D). In contrast, treatment of cells with 100 μM EDTA had no effect on tumour cell apoptosis when

**Table 1** Comparison of the effect of zoledronic acid and EDTA on MCF-7 cell number and apoptosis

| Treatment[a] | Cell number ($\times 10^4$)[b] | Apoptosis (%) |
|---|---|---|
| Control | 24.52 ± 4.3 | 1.09 ± 0.5 |
| EDTA (100 μM) | 16.08 ± 4.2* | 1.30 ± 0.5 |
| Zoledronic acid (100 μM) | 7.00 ± 1.5*† | 8.60 ± 1.2* |

[a]Cells were incubated with control, zoledronic acid or EDTA for a total of 72 hours. [b]Results are expressed as mean ± SEM ($n$ = 4). *$P < 0.05$ compared with control. † $P < 0.05$ compared with EDTA.

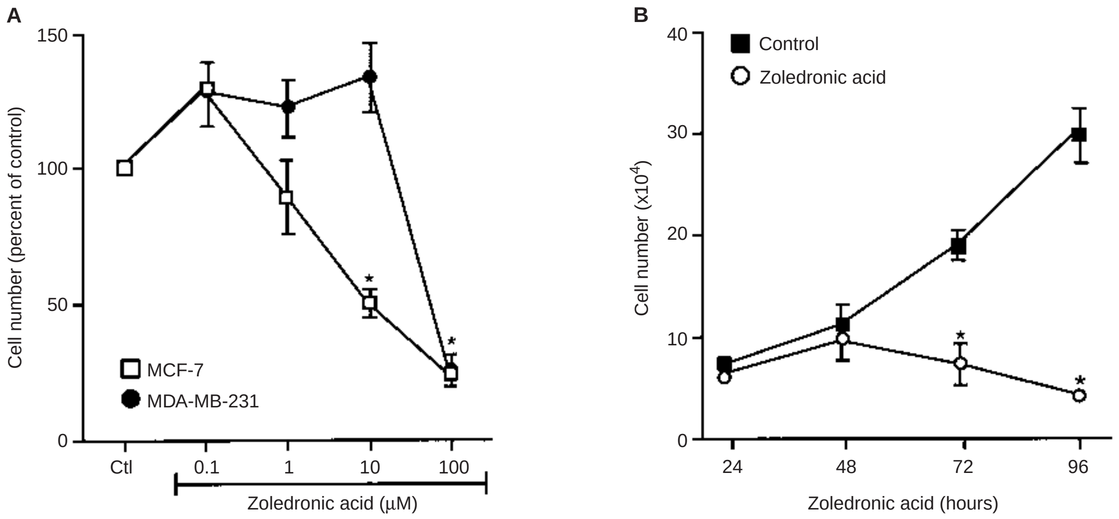

**Figure 1** Effect of (**A**) increasing concentrations of zoledronic acid (0.1–100 μM) on MCF-7 and MDA-MB-231 cell number after incubation for 72 hours and (**B**) increasing time of incubation of MCF-7 cells (24–96 hours) with 100 μM zoledronic acid. Results are expressed as percent of control or absolute cell numbers. Data are the mean and SEM ($n$ = 4). *$P < 0.05$ compared with control

 *© 2001 Cancer Research Campaign*

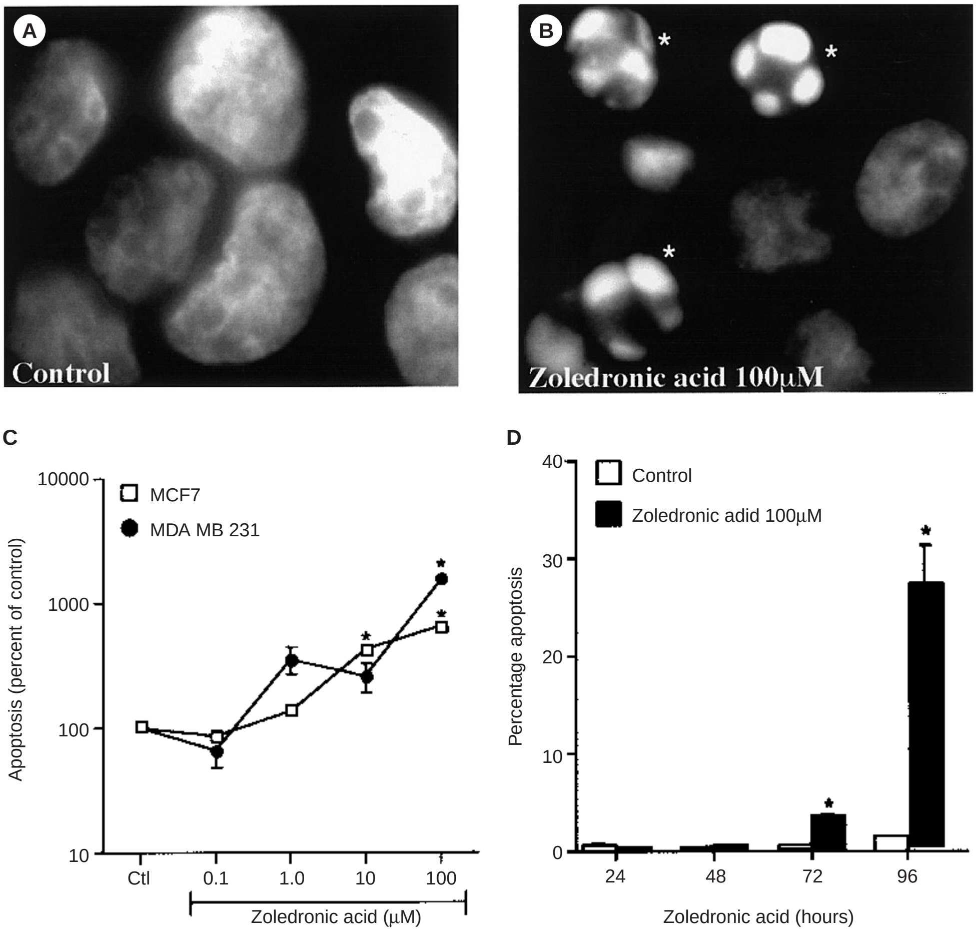

**Figure 2** Effect of zoledronic acid on apoptosis of MCF-7 and MDA-MB-231 breast cancer cells. Fluorescence micrographs of MCF-7 cells incubated with (**A**) vehicle control and (**B**) 100 μM zoledronic acid for 72 hours demonstrating characteristic changes in nuclear morphology. Cells were cytospun, stained with 4′-6-diamidino-2-phenyl-indole (DAPI) and examined with a × 100 oil immersion objective. Apoptotic nuclei are identified with an asterisk. The effect of (**C**) increasing concentrations of zoledronic acid (0.1–100 μM) on MCF-7 and MDA-MB-231 cell apoptosis after 72 hours treatment with 100 μM zoledronic acid. The effect of increasing time of incubation (24–96 hours) with 100 μM zoledronic acid on MCF-7 apoptosis are shown in panel **D**. Results are expressed as percent of the appropriate control or as absolute percentages. Data are the mean ± SE ($n$ = 4). *$P$ < 0.05 compared with control.

compared to control (1.3 ± 0.5% vs 1.09 ± 0.5% respectively) (Table 1).

The induction of apoptosis in MCF-7 and MDA-MB-231 cells by zoledronic acid was confirmed using a fluorescence in situ nick translation assay. Treatment of MCF-7 cells with a range of zoledronic acid concentrations had little effect on apoptosis at 0.1 and 1.0 μM, however, an increase in the proportion of apoptotic cells was observed with 10 μM and 100 μM zoledronic acid compared with control (28.7% and 70.7% vs 22.57%, respectively). Treatment of MDA-MB-231 cells with 0.1–1 μM zoledronic acid did not cause an increase in apoptosis although treatment with the 10 and 100 μM zoledronic acid resulted in a significant increase in the proportions of apoptotic cells (126.6% and 126.6% of control) (data not shown). A significant time-dependent increase in MCF7 cell apoptosis was confirmed when cells were incubated with 100 μM zoledronic acid for 24–96 hours (Table 2).

**Table 2** The effects of 100 μM zoledronic acid on MCF-7 cell apoptosis after 24, 48, 72 and 96 hours of incubation

| Time (hours) | Control (%)[a] | Zoledronic acid (%)[a] |
|---|---|---|
| 24 | 19.3 | 10.4 |
| 48 | 8.6 | 24.47 |
| 72 | 22.67 | 48.93 |
| 96 | 14.57 | 33.07 |

[a]Apoptosis was assessed by the in situ nick translation assay and results were expressed as percentages.

### Induction of apoptosis of breast cancer cells by zoledronic acid occurs via inhibition of enzymes of the mevalonate pathway

The effects of the addition of intermediates of the mevalonate pathway on zoledronic acid-induced MCF-7 cell apoptosis were determined. Analysis of nuclear morphology demonstrated that treatment of MCF-7 cells with 100 μM zoledronic acid resulted in a significant increase in apoptosis (18.68 ± 2.5%) compared with control (0.26 ± 0.1%). The addition of 50 μM farnesol resulted in a partial inhibition of apoptosis (14.31 ± 0.3%), whereas the addition of 50 μM geranylgeraniol inhibited apoptosis to levels close to control (2.27 ± 0.2%) (data not shown). These effects were confirmed using the in situ nick translation assay (Figure 3).

*© 2001 Cancer Research Campaign*

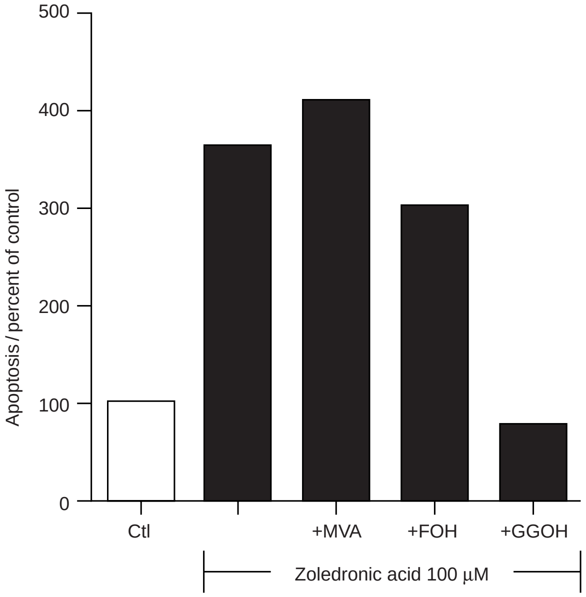

**Figure 3** Effects of intermediates of the mevalonate pathway on zoledronic acid-induced apoptosis of MCF-7 cells. Mevalonate and farnesol have little effect on zoledronic acid-induced apoptosis whereas 50 μM geranylgeraniol rescues MCF-7 cells from apoptosis induced by zoledronic acid. Results are expressed as percent of control

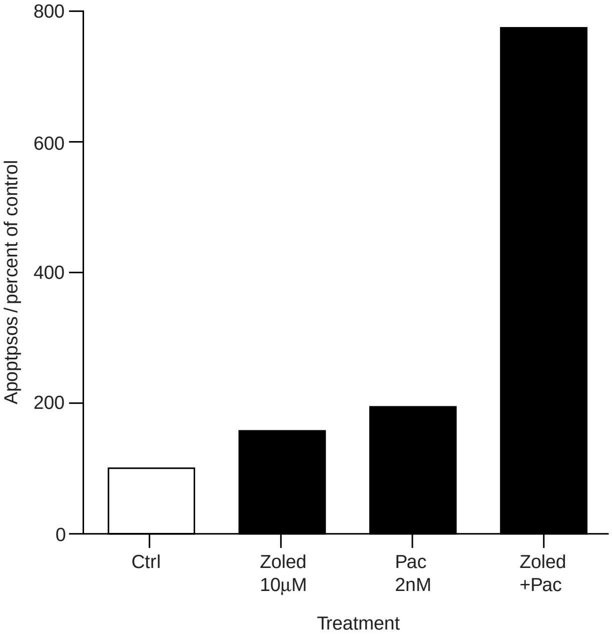

**Figure 4** Effect of combined treatment of MCF-7 cells with 10 μM zoledronic acid and 2 nM paclitaxel. Cells were incubated with 10 μM zoledronic acid in the presence or absence of 2 nM paclitaxel for a total of 72 hours. Apoptosis was assessed using the in situ nick translation assay. Results are expressed as percent of control

Treatment of MCF-7 cells with 100 μM zoledronic acid resulted in a significant increase in apoptosis compared to control (60.57% and 16.6% apoptosis, respectively). The addition of 100 μM mevalonate or 50 μM farnesol to zoledronic acid had little effect on zoledronic acid-induced apoptosis (54.83% and 49.17% respectively). In contrast, the addition of 50 μM geranylgeraniol inhibited zoledronic acid-induced apoptosis to levels below that of control (12.11%).

### Combined treatment with zoledronic acid and paclitaxel, at pharmacologically achievable concentrations, results in synergistic effects on breast cancer cell number and apoptosis

In preliminary studies, we investigated the effect of zoledronic acid in combination with paclitaxel on apoptosis of MCF-7 cells. Analysis of nuclear morphology demonstrated that combining zoledronic acid and paclitaxel caused a greater than 2-fold increase in the proportion of apoptotic MCF-7 cells when compared with either drug alone ($P < 0.02$) (data not shown). The fluorescence DNA labelling assay revealed that treatment with 10 μM zoledronic acid and 2 μM paclitaxel resulted in a 5-fold increase in apoptosis (774.8% of control) compared to zoledronic acid alone (155.71%) and a 4-fold increase compared to paclitaxel alone (189.68%) (Figure 4). Since these data suggest that these compounds may have synergistic effects, a more detailed isobologram analysis was undertaken.

The combination of paclitaxel and zoledronic acid was shown to result in synergistic effects on MCF-7 cell number and apoptosis (Figure 5(I) and (II)). Dose–response curves were plotted for the effect of paclitaxel (IA) and zoledronic acid (IB) on cell number and the $IC_{75}$ values calculated. The resulting data points were plotted on an isobologram (IC). The D/D75 ratios for paclitaxel alone (1) and zoledronic acid alone (1) are shown and connected by a diagonal line. Points lying on this line indicate a purely additive effect. Data points forming a curve lying above this line indicate antagonism between the 2 drugs whereas a curve lying below this line suggests a synergistic interaction. The combined D/D75 ratios derived from the dose–response data clearly formed a curve which lay below the additive effect line, indicating synergistic effects between paclitaxel and zoledronic acid on tumour cell number.

Combined treatment of MCF-7 cells with paclitaxel and zoledronic acid was shown to result in a synergistic increase in tumour cell apoptosis (Figure 5(II)). Combined drug $IC_5$ values were determined from dose–response curves plotted for paclitaxel (IIA) and zoledronic acid (IIB). These were then used to construct an isobologram (IIC) as described above. Again, the combined D/D5 ratios, when plotted on the isobologram, formed a curve that lay below the additive effect line indicating a synergistic effect on tumour cell apoptosis.

### Acute exposure to zoledronic acid causes a reduction in breast cancer cell number and an increase in apoptosis

Zoledronic acid inhibits breast cancer cell proliferation and induces apoptosis in vitro in a dose- and time-dependent fashion. However in vivo, breast cancer cells are probably exposed to micromolar concentrations of bisphosphonates for only a few hours. Therefore, in order to look at conditions that may more accurately reflect the situation in vivo, we examined the effects of acute exposure to 100 μM zoledronic acid on MCF-7 cell number and apoptosis (Figure 6). 2 hours of exposure to zoledronic acid

© 2001 Cancer Research Campaign

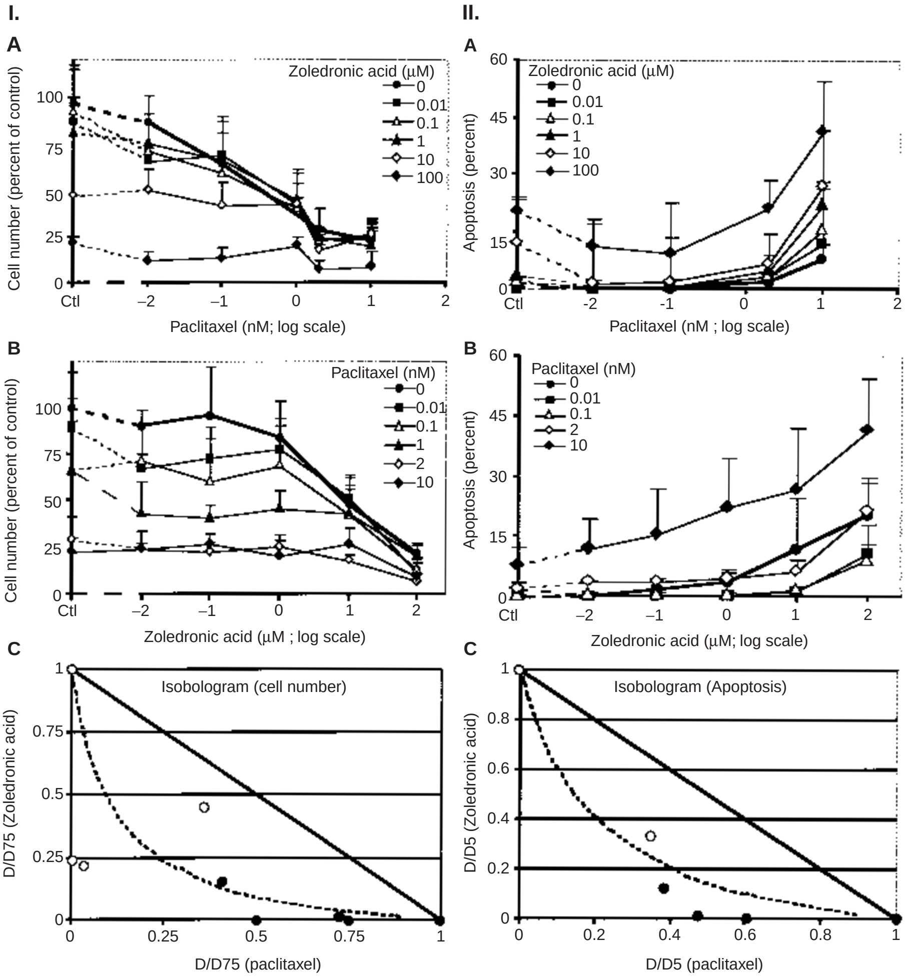

**Figure 5** Dose–response curves and isobologram plots for the interaction between paclitaxel and zoledronic acid on MCF7 cell number (**I**) and apoptosis (**II**). Cells were incubated with increasing concentrations of zoledronic acid (0.01–100 μM) in combination with increasing concentrations of paclitaxel (0.01–10 nM) for a total of 72 hours. Cells were harvested to determine cell number ($n$ = 4) and dose–response curves were plotted for effect of paclitaxel (**IA**) and zoledronic acid (**IB**) on cell number. Combined drug $IC_{75}$ values were determined from these data and used to construct an isobologram (**IC**) for the interaction between paclitaxel (●) and zoledronic acid (○). Apoptosis was determined using changes in nuclear morphology ($n$ = 4) and dose–response curves were plotted for effect of paclitaxel (**IIA**) and zoledronic acid (**IIB**) on apoptosis. Combined drug $IC_5$ values were determined from these data and used to construct an isobologram (**IIC**) for the interaction between paclitaxel (●) and zoledronic acid (○). Dose–response results were expressed as percent of control and are means ± SE.

resulted in reduction in cell number to 77.1 ± 19% of control ($P < 0.05$). This was associated with an increase in the proportions of apoptotic cells from 1.23 ± 0.5% in the control samples to 8.05 ± 0.6% in zoledronic acid-treated cells ($P < 0.05$). 6 and 24 hours of exposure resulted in a reduction in cell number of 44.75 ± 7.9% and 30.04 ± 9.8% of control, respectively ($P < 0.05$). The proportions of apoptotic cells at the 6 and 24 hour time points were similar to that at 2 hours (8.24 ± 0.7% and 8.24 ± 1.1% respectively) ($P < 0.05$) indicating that acute exposure to zoledronic acid results in significant induction of apoptosis. The induction of apoptosis by zoledronic acid at 2, 6 and 24 hours was confirmed using the in situ nick translation assay. 2 hours of exposure to 100 μM zoledronic acid showed an increase in apoptosis of 236.03% of control. This proportion increased to 289.15% after 6 hours of exposure and 643.62% after 24 hours (values expressed as percent of appropriate controls).

### DISCUSSION

In the present study we have shown that zoledronic acid has a dose-and time-dependent effect on breast cancer cell number in vitro. These observations are consistent with previous studies that have demonstrated that bisphosphonates cause a significant reduction in osteoclast number (Hughes et al, 1995; Fisher et al, 1999). Importantly, studies in human myeloma cells have shown that nitrogen-containing bisphosphonates, including zoledronic acid, also inhibit cell proliferation (Shipman et al, 1997, 1998; Aparicio et al, 1998; Derenne et al, 1999). The effects of zoledronic acid on MCF-7 and MDA-MB-231 cell number were associated with concomitant dose- and time-dependent changes in nuclear morphology that were characteristic of apoptosis (Wyllie et al, 1980). These effects on tumour cell apoptosis are consistent with previous reports that have demonstrated the induction of apoptosis, in macrophages, osteoclasts and myeloma cells, by

© 2001 Cancer Research Campaign

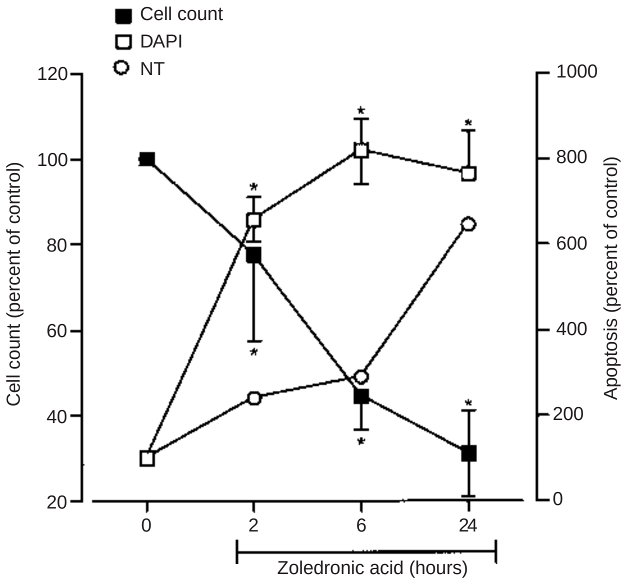

**Figure 6** Effect of short-term exposure of MCF-7 cells to 100 μM zoledronic acid. MCF-7 cells were incubated with zoledronic acid for 2, 6, 24 and 72 hours. The media was then replaced and the cells were maintained for a total of 72 hours. The effects on cell number (*n* = 4) and apoptosis as assessed by in situ nick translation assay (NT) and changes in nuclear morphology (DAPI, *n* = 4) are shown. Results are expressed as percent of control and are the mean ± SE. $*P < 0.05$ compared with contro.l bisphosphonates such as pamidronate, incadronate, and zoledronic acid (Hughes et al, 1995; Rogers et al, 1996; Shipman et al, 1997, 1998; Aparicio et al, 1998; Fisher et al, 1999). Although there were significant differences between the levels of apoptosis detected by the 2 different techniques, the relative changes induced by bisphosphonate treatment were similar. The reasons for the discrepancy between methods are unclear but may reflect the conservative assessment of nuclear morphology. Alternatively, the fluorescence in situ nick translation assay may be identifying cells in an earlier stage of apoptosis.

In the present study concentrations of 10–100 μM zoledronic acid for 72 h were required to induce apoptosis of breast cancer cells. However, bisphosphonates have a high affinity for mineralized bone and localize to bone rapidly, thus in a clinical setting it is likely that primary breast cancer cells or those in visceral metastases may be exposed to these compounds for shorter periods. Indeed, peak serum concentrations are likely to be in the range of 1–3 μM and maintained for only a few hours (Berenson et al, 2000). In bone metastases, where osteoclasts are resorbing bone loaded with bisphosphonate, and thereby releasing it, prolonged exposure to high local concentrations of the compound would be predicted (Sato et al, 1991). However, exposure times of 2, 6 and 12 hours may more accurately reflect those seen by cells other than osteoclasts in vivo. Incubation of MCF-7 cells with 100 μM zoledronic acid for these periods resulted in a similar reduction in cell number and increase in apoptosis, suggesting that acute exposure is sufficient to have an anti-tumour effect in these cells. This is consistent with observations from a study in J774 macrophages which demonstrated a time-dependent increase in caspase-3-like enzyme activity, associated with apoptosis, when acutely exposed to nitrogen-containing bisphosphonates (Coxon et al, 1998).

Importantly, in the clinical arena, bisphosphonates are also used in combination with chemotherapy or endocrine therapy (Diel et al, 1998). Also, studies in animal models of breast cancer suggest that the anti-tumour activity of bisphosphonates may be enhanced when they are combined with chemotherapeutic agents (Yoneda et al, 2000). The present study has shown that treatment of MCF-7 cells with zoledronic acid and 2 nM paclitaxel, a concentration readily achievable in the serum of patients (Wilson et al, 1994), results in an increase in apoptosis above the expected additive effect. To investigate this more fully and to demonstrate synergy, the isobologram method was used in our studies. This method allows for the analysis of the interaction between 2 drugs and has been used both in vitro and in vivo (Gessner, 1988; Leonessa et al, 1994). Our dose–response studies and isobologram analysis showed evidence of synergistic action between paclitaxel and zoledronic acid on tumour cell number and apoptosis. The mechanism by which this occurs was not determined but inhibition of the mevalonate pathway due to zoledronic acid treatment and the prevention of chromosome segregation and cell division by paclitaxel may result in a cumulative apoptotic insult. These mechanisms require further study both in vitro and in vivo. It would also be important to investigate possible synergy with other anti-neoplastic agents, as well as to explore temporal relationships, in future studies.

Although zoledronic acid clearly has cytotoxic effects on breast cancer cells, these may be mediated by their ability to chelate calcium. However, studies with EDTA, which will also chelate calcium, demonstrated that this has only a small effect on the reduction in cell number and remained significantly different from the effect observed following zoledronic acid treatment. Unlike the effect on cell number, EDTA treatment did not result in a significant increase in apoptosis compared to control suggesting that the apoptosis induced by zoledronic acid is not mediated by chelation of calcium. The reasons for the differences in these effects on cell number and apoptosis are unclear; however, calcium chelation with EDTA may be having cytostatic effect on breast cancer cells rather than a cytotoxic effect. Interestingly, MCF-7 and MDA-MB-231 cells showed different levels of sensitivity to zoledronic acid, the reasons for which remain unclear. However, this may reflect the differing expression of *p53* as MCF-7 cells express wild-type *p53* whilst MDA-MB-231 cells express a mutant *p53* sequence, which may confer increased resistance to apoptotic stimuli (O'Connor et al, 1997). Alternatively, MDA-MB-231 cells may produce a number of cytokines or local growth factors that could act as survival factors and inhibit zoledronic acid-induced apoptosis (Fromigue and Body, 2000). There may also be differences, between the cell lines, in the way in which zoledronic acid is taken up.

We have also demonstrated that zoledronic acid-induced apoptosis of MCF-7 breast cancer cells can be inhibited by addition of intermediates of the mevalonate pathway, which is consistent with observations in osteoclasts, macrophages and myeloma cells (Luckman et al, 1998; Shipman et al, 1998; Fisher et al, 1999). This pathway has been shown to be important in the post-translational modification of small GTP-binding proteins including Ras, by the process of farnesylation, and Rho and Rac by geranylgeranylation. These proteins are involved in a range of important cellular processes including proliferation (Ras), membrane trafficking and cytoskeletal organization (Rho and Rac). In the present study, the addition of geranylgeraniol, rescued

 © 2001 Cancer Research Campaign

MCF-7 cells from zoledronic acid-induced apoptosis, whereas the addition of farnesol only partially inhibited tumour cell death. Recent observations by Fisher et al suggest that this may also be the case in osteoclasts where disruption of the cytoskeleton and loss of the osteoclast ruffled border, as well as apoptosis, are seen with bisphosphonate treatment (Fisher et al, 1999). However, this contrasts with previous studies in J774 macrophages and the JJN3 myeloma cells in which bisphosphonate-induced apoptosis could be prevented by the addition of both geranylgeraniol and farnesol. The reasons for the differences between these studies are unclear; however, farnesylated proteins such as Ras may be less important in breast cancer cell and osteoclast function than in macrophages and myeloma cells.

In summary, the data presented in this study strongly suggest that zoledronic acid can have an important anti-tumour effect in breast cancer cells in vitro by promoting tumour cell apoptosis. It is therefore possible that the beneficial effects seen in patients receiving bisphosphonate therapy may be due to anti-tumour effects as well as inhibition of osteoclast-mediated bone resorption. These observations warrant further investigation in vivo in patients receiving bisphosphonates.

### ACKNOWLEDGEMENTS

These studies were supported by Yorkshire Cancer Research and Novartis Pharma AG. Dr J Lawry and Mrs O Smith at the YCR Institute of Cancer Studies provided support with flow cytometry analysis.

### REFERENCES

Aparicio A, Gardner A, Tu Y, Savage A, Berenson J and Lichtenstein A (1998) *In vitro* cytoreductive effects on multiple myeloma cells induced by bisphosphonates. *Leukaemia* **12**: 220–229

Berenbaum M (1989) What is synergy? *Pharmacol Rev* **41**: 93–141

Berenson J, Lichtenstein A, Porter L, Dimopoulos M, Bordoni R, George S, Lipton A, Keller A, Ballester O, Kovacs M, Blacklock H, Bell R, Simeone J, Reitsma D, Heffernan M, Seaman J and Knight R (1996) Efficacy of pamidronate in reducing skeletal events in patients with advanced multiple myeloma. *New Engl J Med* **334**: 488–493

Berenson JR, Ravera C, Ma P, Deckert F, Sasaki Y, Saeki T, Takashima S, LoRusso P, Goodin S, Seaman J, Schran H and Zhou H (2000) Population Pharmacokinetics (PK) of Zometa. *Proc ASCO*

Boissier S, Magnetto S, Frappart L, Cuzin B, Ebetino FH, Delmas, PD and Clezardin P (1997) Bisphosphonates inhibit prostate and breast carcinoma cell adhesion to unmineralized and mineralized bone extracellular matrices. *Cancer Res*, **57**: 3890–3894

Coxon FP, Benford HL, Russell RG and Rogers MJ (1998) Protein synthesis is required for caspase activation and induction of apoptosis by bisphosphonate drugs. *Mol Pharmacol* **54**: 631–638

Derenne S, Amiot M, Barille S, Collette M, Robillard N, Berthaud P, Harousseau JL and Bataille R (1999) Zoledronate is a potent inhibitor of myeloma cell growth and secretion of IL-6 and MMP-1 by the tumoral environment. *J Bone Miner Res* **14**: 2048–56

Diel IJ, Solomayer EF, Costa SD, Gollan C, Goerner R, Wallwiener D, Kaufmann M and Bastert G (1998) Reduction in new metastases in breast cancer with adjuvant clodrnate treatment. *New Engl J Med* **339**: 357–363

Fisher JE, Rogers MJ, Halasy JM, Luckman SP, Hughes DE, Masarachia PJ, Wesolowski G, Russell RG, Rodan GA and Reska AA (1999) Alendronate mechanism of action: geranylgeraniol, an intermediate in the mevalonate pathway, prevents inhibition of osteoclast formation, bone resorption, and kinase activation in vitro. *Proc Natl Acad Sci USA* **96**: 133–8

Frith JC, Monkkonen J, Blackburn GM, Russell RG and Rogers MJ (1997) Clodronate and liposome-encapsulated clodronate are metabolized to a toxic ATP analogue, adenosine 5′-(beta, gamma-dichloromethylene) triphosphate, by mammalian cells in vitro. *J Bone Miner Res* **12**: 1358–67

Fromigue O and Body J (2000) Bisphosphonates induce breast cancer cell death *in vitro*. *J Bone Miner Res* **15**: 2211–2221

Gessner, P (1988) A straightforward method for the studyof drug interactions: an isobolographic primer. *J Am Coll Toxicol* **7**: 987–1012

Gorczyca W, Myron R, Melamed and Darzynkiewicz Z (1993) Apoptosis of S-phase HL-60 cells induced by DNA topoisomerase inhibitors: Detection of DNA strand breaks by flow cytometry using the *in situ* nick translation assay. *Toxicol Lett* **67**: 249–258

Greco WR, Bravo G and Parsons J (1995) The search for synergy: A critical review from a response surface perspective. *Pharmacol Rev* **47**: 331–385

Hortobagyi GN, Theriault RL, Porter L, Blayney D, Lipton A, Sinoff C, Wheeler H, Simeone JF, Seaman J and Knight RD (1996) Efficacy of pamidronate in reducing skeletal complications in patients with breast cancer and lytic bone metastases. Protocol 19 Aredia Breast Cancer Study Group. *New Engl J Med* **335**: 1785–1791

Hughes DE, Wright KR, Uy HL, Sasaki A, Yoneda T, Roodman GD, Mundy GR and Boyce BF (1995) Bisphosphonates promote apotosis in murine osteoclasts in vitro and in vivo. *J Bone Miner Res* **10**: 1478–1487

Kanis JA, Powles T, Paterson AH, McCloskey EV and Ashley S (1996) Clodronate decreases the frequency of skeletal metastases in women with breast cancer. *Bone* **19**: 663–667

Leonessa F, Jacobson M, Boyle B, Lippman J, McGarvey M and Clarke R (1994) Effect of tamoxifen on the multidrug-resistant phenotype in human breast cancer cells: isobologram, drug accumulation and $M_r$ 170,000 glycoprotein (gp170) binding studies. *Cancer Res* **54**: 441–447

Luckman SP, Hughes DE, Coxon FP, Graham R, Russell G and Rogers MJ (1998) Nitrogen-containing bishosphonates inhibit the mevalonate pathway and prevent post-translational prenylation of GTP-binding proteins, including Ras. *J Bone Miner Res* **13**: 581–589

Magnetto S, Boissier S, Delmas PD and Clezardin P (1999) Additive anti-tumour activities of taxoids in combination with the bisphosphonate ibandronate against invasion and adhesion of human breast carcinoma cells to bone. *Int J Cancer* **83**: 263–269

McCloskey EV, Maclennan CM, Drayson MT, Chapman C, Dunn J and Kanis JA (1998) A randomized trial of the effect of clodronate on skeletal morbidity in multiple myeloma. *Br J Haematol* **100**: 317–325

O'Connor PM, Jackman J, Bae I, Myers TG, Fan S, Mutoh M, Scudiero DA, Monks A, Sausville EA, Weinstein JN, Friend S, Fornace AJ Jr and Kohn KW (1997) Characterization of the p53 tumour suppressor pathway in cell lines of the National Cancer Institute anticancer drug screen and correlations with the growth- inhibitory potency of 123 anticancer agents. *Cancer Res* **57**: 4285–4300

Paterson AHG, Powles TJ, Kanis JA, McCloskey E, Hanson J and Ashley S (1993) Double-blind controlled trial of oral clodronate in patients with bone metastases from breast cancer. *J Clin Oncol* **11**: 59–65

Rogers M, Chilton K, Coxon F, Lawry J, Smith M, Suri S and Russell R (1996) Bisphosphonates induce apoptosis in mouse macrophage-like cells in vitro by a nitric oxide-independent mechanism. *J Bone Miner Res* **11**: 1482–1491

Saarto T, Blomqvist C, Virkkunen P and Elomaa I (2000) Adjuvant clodronate does not reduce the frequency of skeletal metastases in node-positive breast cancer patients. *J Clin Oncol* **19**: 10–19

Sato M, Grasser W, Endo N, Akins R, Simmons H and Thompson DD (1991) Bisphosphonate action. Alendronate localisation in rat bone and effects on osteoclast ultrastructure. *J Clin Invest* **88**: 2095–2105

Shipman CM, Rogers MJ, Apperley JF, Russell RG and Croucher PI (1997) Bisphosphonates induce apoptosis in human myeloma cell lines: a novel anti-tumour activity. *Br J Haematol* 665–672

Shipman CM, Croucher PI, Russell RG, Helfrich MH and Rogers MJ (1998) The bisphosphonate incadronate (YM175) causes apoptosis of human myeloma cells in vitro by inhibiting the mevalonate pathway. *Cancer Res* **58**: 5294–5297

Theriault RL, Lipton A, Hortobagyi GN, Leff R, Gluck S, Stewart JF, Costello S, Kennedy I, Simeone J, Seaman JJ, Knight RD, Mellars K, Heffernan M and Reitsma DJ (1999) Pamidronate reduces skeletal morbidity in women with advanced breast cancer and lytic bone lesions: a randomized, placebo-controlled trial. Protocol 18 Aredia Breast Cancer Study Group. *J Clin Oncol* **17**: 846–854

Van der Pluijm G, Vloedgraven H, Van Beek E, Van der Wee-Pals L, Lowik C and Papapoulos S (1996) Bisphosphonates inhibit the adhesion of breast cancer cells to bone matrices in vitro. *J Clin Invest* **98**: 698–705

Wilson WH, Berg SL, Bryant G, Wittes RE, Bates S, Fojo A, Steinberg SM, Goldspiel BR, Herdt J and O'Shaughnessy J (1994) Paclitaxel in doxorubicin-

© 2001 Cancer Research Campaign 

refractory or mitoxantrone-refractory breast cancer: a phase I/II trial of 96-hour infusion. *J Clin Oncol* **12**: 1621–1629
Wyllie A, Kerr J and Currie A (1980) Cell death: The significance of apoptosis. *Int Rev Cytol* **68**: 251–306
Yoneda T, Michigami T, Yi B, Williams PJ, Niewolna M and Hiraga T (2000) Actions of bisphosphonate on bone metastasis in animal models of breast carcinoma. *Cancer* **88**: (12 Suppl), 2979–2988

*© 2001 Cancer Research Campaign*
